# Genome-Wide *Anaplasma phagocytophilum* AnkA-DNA Interactions Are Enriched in Intergenic Regions and Gene Promoters and Correlate with Infection-Induced Differential Gene Expression

**DOI:** 10.3389/fcimb.2016.00097

**Published:** 2016-09-20

**Authors:** J. Stephen Dumler, Sara H. Sinclair, Valeria Pappas-Brown, Amol C. Shetty

**Affiliations:** ^1^Department of Pathology, F. Edward Hébert School of Medicine, Uniformed Services University of the Health SciencesBethesda, MD, USA; ^2^Science Division, Dine CollegeTsaile, AZ, USA; ^3^Informatics Resource Center, Institute for Genome Sciences, University of MarylandBaltimore, MD, USA

**Keywords:** AnkA, *Anaplasma phagocytophilum*, matrix attachment regions, cellular reprogramming, MHC locus, nuclear lamina

## Abstract

*Anaplasma phagocytophilum*, an obligate intracellular prokaryote, infects neutrophils, and alters cardinal functions via reprogrammed transcription. Large contiguous regions of neutrophil chromosomes are differentially expressed during infection. Secreted *A. phagocytophilum* effector AnkA transits into the neutrophil or granulocyte nucleus to complex with DNA in heterochromatin across all chromosomes. AnkA binds to gene promoters to dampen *cis*-transcription and also has features of matrix attachment region (MAR)-binding proteins that regulate three-dimensional chromatin architecture and coordinate transcriptional programs encoded in topologically-associated chromatin domains. We hypothesize that identification of additional AnkA binding sites will better delineate how *A. phagocytophilum* infection results in reprogramming of the neutrophil genome. Using AnkA-binding ChIP-seq, we showed that AnkA binds broadly throughout all chromosomes in a reproducible pattern, especially at: (i) intergenic regions predicted to be MARs; (ii) within predicted lamina-associated domains; and (iii) at promoters ≤ 3000 bp upstream of transcriptional start sites. These findings provide genome-wide support for AnkA as a regulator of *cis*-gene transcription. Moreover, the dominant mark of AnkA in distal intergenic regions known to be AT-enriched, coupled with frequent enrichment in the nuclear lamina, provides strong support for its role as a MAR-binding protein and genome “re-organizer.” AnkA must be considered a prime candidate to promote neutrophil reprogramming and subsequent functional changes that belie improved microbial fitness and pathogenicity.

## Introduction

Obligately intracellular bacteria, such as *Anaplasma phagocytophilum*, evolved mechanisms for survival within, subversion of host functions, or symbiotic relationships with their eukaryotic hosts. While tick-transmitted *A. phagocytophilum* infection results in clinical disease manifestations in some animals and humans, referred to as granulocytic anaplasmosis, its mere occupancy in the most abundant host defense cell, the neutrophil, is an intriguing evolutionary adaptation, owing to its role in innate immunity where it recognizes and kill microbes (Scapini and Cassatella, 2014). Once within the neutrophil, *A. phagocytophilum* survives long enough for continued replication and subsequent transmission via tick bite (Rikihisa, 2011; Dumler, 2012; Truchan et al., 2013). Major alterations induced by *A. phagocytophilum* infection of human neutrophils impact antimicrobial responses such as respiratory burst, phagocytosis, margination, and emigration across the endothelium, delayed apoptosis and increased production of proinflammatory cytokines, chemokines, and proteases (Carlyon et al., 2002; Carlyon and Fikrig, 2003, 2006; Choi et al., 2005; Dumler et al., 2005; Garyu et al., 2005).

How changes in host cell function occur is partly explained by studies of gene transcription among infected host granulocytes (Borjesson et al., 2005; de la Fuente et al., 2005; Lee et al., 2008). Downregulation of genes encoding two NADPH components, gp91^*phox*^ encoded by *CYBB*, and the GTPase RAC2, represses superoxide generation by respiratory burst to inhibit bacterial killing (Banerjee et al., 2000; Carlyon et al., 2002). Similarly, sustained transcription of *BCL2* family member genes that repress apoptosis results in prolonged survival of infected neutrophils (Choi et al., 2005; Ge et al., 2005). In addition, infection is strongly associated with increased transcription from a number of cytokine and chemokine genes, including *IL1A* and *CXCL8* (IL-8), that contribute to recruitment of new neutrophil hosts, and to inflammatory tissue injury and disease (Klein et al., 2000; Akkoyunlu et al., 2001; Scorpio et al., 2004).

We previously showed that *A. phagocytophilum* infection leads to altered chromatin structure at the promoters of many host defense genes driven by AnkA binding (Garcia-Garcia et al., 2009a,b). AnkA binding at promoters recruits HDAC-1 to deacetylate histone H3, leading to the exclusion of RNA polymerase (Rennoll-Bankert et al., 2015). Gene silencing by *cis*-binding of AnkA does not explain upregulated gene expression or the multiple transcriptional changes, including those that are coordinated for reprogramming of a host cell. In this light, we also showed that transfection of AnkA alone, a type IV system secretion substrate, mimics many transcriptional changes observed with infection, including those associated with direct binding to host DNA in many genomic regions (Garcia-Garcia et al., 2009b). The genome-wide binding of AnkA is linked to the observation that it binds DNA in a sequence-independent manner at base-unpairing regions—regions characterized by long stretches of A, T, and C nucleotides on a single strand that lead to uncoiling under superhelical pressure. These regions are also known as matrix attachment regions (MARs) because they bind proteins that enable 3-dimensional architecture changes in chromatin and facilitate long-range interactions critical for transcriptional reprogramming events (Yokota and Kanakura, 2014). Dramatic restructuring of chromatin architecture in coordinated reprogramming events can occur with *A. phagocytophilum* infection as demonstrated by transcriptional profiling of infected human neutrophils *ex vivo* where multiple long contiguous genome regions are differentially regulated over all 23 chromosomes (Borjesson et al., 2005; Sinclair et al., 2014, 2015). Whether AnkA binding affects 3-dimensional structure of the neutrophil genome and whether this relates to differential gene expression from these genomic regions are important questions. Herein, we describe the genome-wide distribution of AnkA reacted with DNA from *ex vivo* human neutrophils, their specific binding sites, how binding and enrichment correlate with different transcriptional regulation mechanisms, and its potential impact on chromosomal regions with extensive differential gene transcription compared to uninfected cells. The data here provide evidence of a direct relationship between these events at the chromosome 6 *MHC* locus, a “proof-of-concept” model site for differential gene transcription.

## Materials and methods

### *Ex vivo* human peripheral blood genomic DNA preparation

Primary peripheral blood neutrophils were isolated from venous blood of 3 healthy adult donors (1 female, 2 males) as approved by the University of Maryland, Baltimore IRB and as previously described (Rennoll-Bankert et al., 2014). Briefly, EDTA anticoagulated blood was dextran-sedimented and leukocyte-rich plasma centrifuged though a Ficoll-Paque gradient. Mononuclear cells were removed and the remaining erythrocytes were lysed in hypotonic saline. Genomic DNA was prepared using a QIAmp DNA Blood Mini Kit (Qiagen).

### Preparation of recombinant AnkA-flag fusion protein

Full-length *ankA* coding sequences were amplified from *A. phagocytophilum* Webster strain genomic DNA and cloned into the pT7-FLAG-MAT-Tag-2 (Sigma-Aldrich) plasmid for expression in *E. coli*. Recombinant AnkA-FLAG was purified on anti-FLAG magnetic beads (GE Healthcare) and quantified. Recombinant FLAG epitope-expressing AnkA was found to be >90% purified by SDS-PAGE and is able to gel-shift the control base unpairing region in the *CYBB* proximal promoter, an established binding site.

### AnkA-neutrophil genomic DNA interactions

To examine genomic binding sites of AnkA, we followed the procedure of Kohwi-Shigematsu (Kohwi-Shigematsu et al., 2012) with modifications as follows: 100 μL reactions containing 5 nM AnkA-FLAG, 10 μg genomic DNA, 1 mg/mL BSA, and 10 μg/mL poly dI-dC in 10 mM HEPES-NaOH, pH 7.9, 50 mM KCl, 2.5 mM Mg_2_Cl, and 1 mM DTT, were incubated at 25⋅C for 25 min. FLAG-magnetic beads (Sigma-Aldrich) were pre-blocked with 10 μg/mL poly dI-dC in 100 mM HEPES-NaOH, pH 7.9, 50 mM KCl, 2.5 mM Mg2Cl, 1 mM DTT, 100 mM imidazole (pH 8.0; binding buffer) for 1 h at 25⋅C. These magnetic beads were used to capture AnkA-FLAG by incubation at 25⋅C for 5 min, then washed 3 times in binding buffer. The captured AnkA-FLAG-DNA complexes were then eluted from the beads in 100 mM HEPES-NaOH, pH 7.9, 100 mM imidazole (pH 8.0), 10 mM EDTA, 1% SDS, and 0.1 mg/mL proteinase K for 5 min at 25⋅C, repeating the elution from the beads once followed by complete digestion at 65⋅C for 1 h. The captured DNA fragments were then purified (QIAquick PCR purification Kit) and sheared by sonication to obtain fragments smaller than 500 bp prior to library construction.

### AnkA-DNA library preparation

Briefly, 5 ng of DNA was end-repaired and adenylated following the manufacturer's protocol (Illumina TruSeq ChIP Sample Preparation Kit). Fragments were then ligated to indexing adapters, purified, and amplified for 18 cycles by PCR. Libraries were assessed for concentration and fragment size using the DNA High Sensitivity Assay on the LabChip GX (Perkin Elmer, Waltham, MA). The library concentrations were also assessed by qPCR using the KAPA Library Quantification Kit (Complete, Universal; Kapa Biosystems, Woburn, MA). The libraries were pooled and sequenced on a 100PE Illumina HiSeq 2500 run (Illumina, San Diego, CA). The sequencing reads were checked for contamination by aligning them to the nucleotide “nt” database using the megablast (a BLAST-like) alignment tool. The quality of the reads was further assessed using the FastQC toolkit to ensure that good quality sequencing reads were utilized for downstream analysis. The reads were aligned to the human genomic reference sequence (Ensembl GRCh38 version) for each of the samples using the BWA (v0.7.12) short-read aligner (Li and Durbin, 2010). The default parameters were used for BWA (“aln -l 25 -t 4”) allowing for 2 mismatches in the seed length of 25 bases. The alignment SAM files generated by BWA were additionally processed to compute binary alignment BAM files and compute the %mapped reads for each of the samples. The total number of reads per sample, the number of mapped reads and the %mapped, and the genome coverage are summarized in Supplementary Table 1. These and all subsequent studies were conducted independently using the 3 individual donors' neutrophils, and all data is shown as a single donor representative of all 3, or as an average of all 3 donor results, as noted.

### Identifying gene regions enriched for AnkA binding

The alignment BAM files were used to identify regions of DNA enrichment using the FindPeaks Peak finder/analysis software (v4.0.16; Fejes et al., 2008). The default parameters were used to identify peaks enriched in the ChIP samples when compared to the input. The peaks were further filtered to retain only those with peak height ≥10, fold enrichment ≥2, and *p* ≤ 0.01 as reported by the software. Figure S1 shows a histogram of peaks for each sample as filtered above. The peaks were then annotated with the overlapping/closest gene using the reference annotation downloaded from Ensembl and the bedtools software toolset. Additionally, the filtered set of peaks and the alignment coverage files were utilized to annotate cis-regulatory elements using the Cis-regulatory Element Annotation System (CEAS) software package (v1.0.2; Shin et al., 2009) to identify potential associations of ChIP regions with functionally important regions such as exons, introns, 5′UTR, 3′UTR, promoters, bi-directional promoters, regions downstream of gene bodies, and distal intergenic regions; this was also used to create average profile analyses of the smoothed adjusted log_2_ (M/T) values across transcriptional start sites (TSS), transcriptional termination sites (TTS), long (2715–11,673 bp), medium (842–2715 bp), and short (158–842 bp) intronic sequences, and long (164–483 bp), medium (109–164 bp), and short (66–109 bp) exonic sequences.

### Identifying chromosomal locations enriched for AnkA-DNA binding and differential gene expression

Publically available transcription microarray data from *A. phagocytophilum*-infected neutrophils was re-analyzed using RMA (GEO accession number GSE2405; Borjesson et al., 2005; Sinclair et al., 2015). Only the 24 h time point was used for comparison with the same interval in the AnkA-binding studies. Differential expression was determined based on the standard deviation of the fold change from the mean. Infected: uninfected fold change gene expression data were transformed to log_2_ for all analyses. Some analyses also used genomic windows that encompass average differential transcription over ~3.9–4.2 Mb (Sinclair et al., 2015). Similarly, AnkA-binding influence over topologically-associated chromatin regions was examined using sliding windows by averaging fold enrichment at each adjacent binding site in the window over stretches (windows) of approximately 53 Mb (IQR 27–94 Mb). In this analysis, each window contains an identical core of individual AnkA binding sites and fold enrichment plotted at the center of the window. The window is then moved one position upstream, excluding the most downstream AnkA binding site in the window and including a new binding site. The result is an average binding fold enrichment over long DNA and chromatin domains approximately the size of recognized topologically-associated domains.

### Identification of AnkA binding at predicted matrix attachment regions (MARs)

AnkA has features of MAR-binding proteins that organize the 3-dimensional structure of chromatin for coordinated transcriptional programs (Garcia-Garcia et al., 2009b; Sinclair et al., 2014; Rennoll-Bankert et al., 2015). To determine whether AnkA binds to MARs, we used the SMARTest Search for S/MARS program (Frisch et al., 2002) adapted for the Genomatix platform (www.genomatix.de). SMARTest scans 300 bp length DNA sequences using a sliding window for matches to the S/MAR matrix library weighted with the AT-rich class of S/MARs. The results of the individual neutrophil DNA-AnkA interactions predicted to bind to a MAR were used to identify those regions using CEAS, as described above.

### Identification of AnkA binding intersections with lincRNA, miRNA, tRNA, and lamina-associated domains

Specific regions of AnkA binding suggested the investigation of intersections with several associated features. These studies were conducted by intersections of datasets using Table Browser in the UCSC Genome Browser. Bedgraph files created from (i) individual AnkA binding sites with fold enrichment and (ii) AnkA binding and average AnkA enrichment over 53 Mb windows (IQR 27–94 Mb) were uploaded to the UCSC Genome Browser. Noncoding RNAs such as long intergenic noncoding RNAs (lincRNAs) and lincRNA gene bed files were obtained from (i) NONCODE (NONCODE2106, www.noncode.org; Zhao et al., 2016), (ii) lincRNA RNA-seq expression ratio tracks (WhiteBloodCell) in the UCSC Genome Browser (Cabili et al., 2011), (iii) Non-coding RNA Super-tracks (lincRNA RNA-seq, lincRNA TUCP [transcripts of uncertain coding potential] expression abundance) (Griffiths-Jones, 2004; Weber, 2005; Griffiths-Jones et al., 2006, 2008), (iv) C/D and H/ACA Box snoRNAs, scaRNAs, and microRNAs (snoRBase version 3 and miRNase Release 13.0), or (v) transfer RNA genes (tRNAscen-SE v.1.23). Because regions of chromosomes that associated with the nuclear lamina have not been established in neutrophils, we estimated potential contacts between AnkA binding sites and the nuclear lamina by constructing a bedgraph file derived from studies on lamina-associated domains of individual clones of the nearly haploid chronic myelogenous leukemia cell line KBM-7 (DataS1_Clone.14.1N.OE; GEO series GSE68263 Clone.14.1N.OE LP150415), where 118 individual clonal cells were evaluated for observed:expected ratios (O:E) of lamina partitioning (Kind et al., 2015). The KBM-7 clone 14 is diploid at chromosome 8 and therefore was not analyzed; it also lacks the Y chromosome. The average O:E at each genomic location over 118 clones was used for the bedgraph file. After uploading to the UCSC Genome Browser, intersections were prepared using Table Browser for statistical evaluation. For some analyses, the correlation function (Pearson's correlation) was used within the Table Browser; Pearson *p*-values were calculated based on n AnkA binding sites or windows and *r*-values. Multiple correlations were evaluated by Student's *t*-test using Bonferroni correction. Where possible, correlations were studied by non-parametric Spearman correlation.

## Results

### Recombinant AnkA-flag binds genome-wide in a reproducible distribution

**Figure 1:**
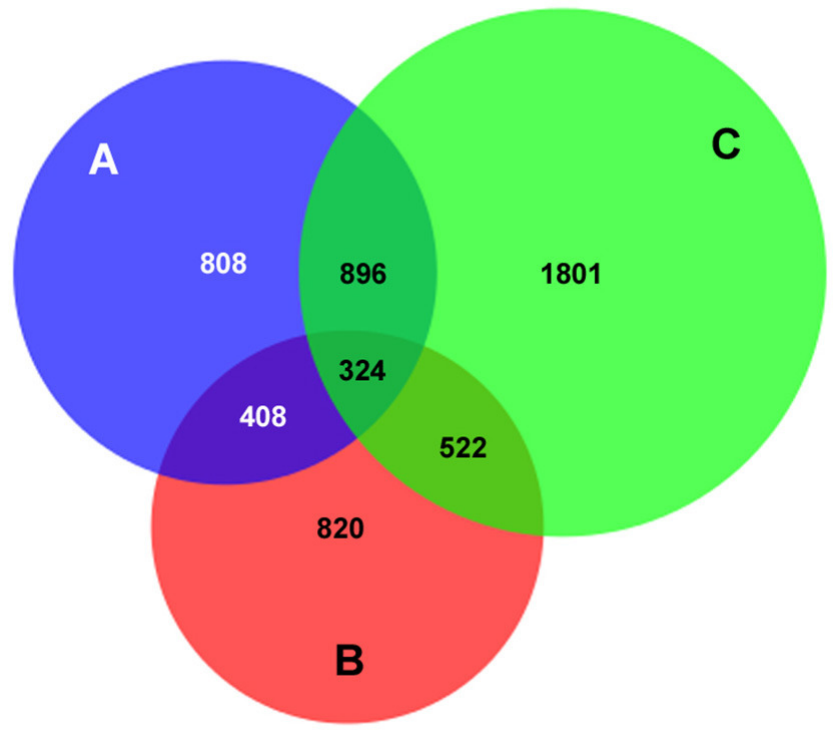
**AnkA binding sites unique to, or in common with those in 3 human neutrophil DNA genome preparations**. The total number of AnkA binding sites for each of the 3 neutrophil DNA preparations after DNA interaction, immunoprecipitation, and sequencing is shown. The Venn diagram demonstrates the core shared among each genome, those shared among 2 genomes, and those uniquely identified in each. For each sequencing reaction, peak calls were identified by FindPeaks and the intersection of these was generated to identify unique or shared binding sites. For ChIP regions of variable length reads, the midpoint was defined (average of the start and end points) and compared to the next closest neighbor assigned by start point. Those that differed by < 100 bp were defined as the same AnkA binding region.

**Figure 2:**
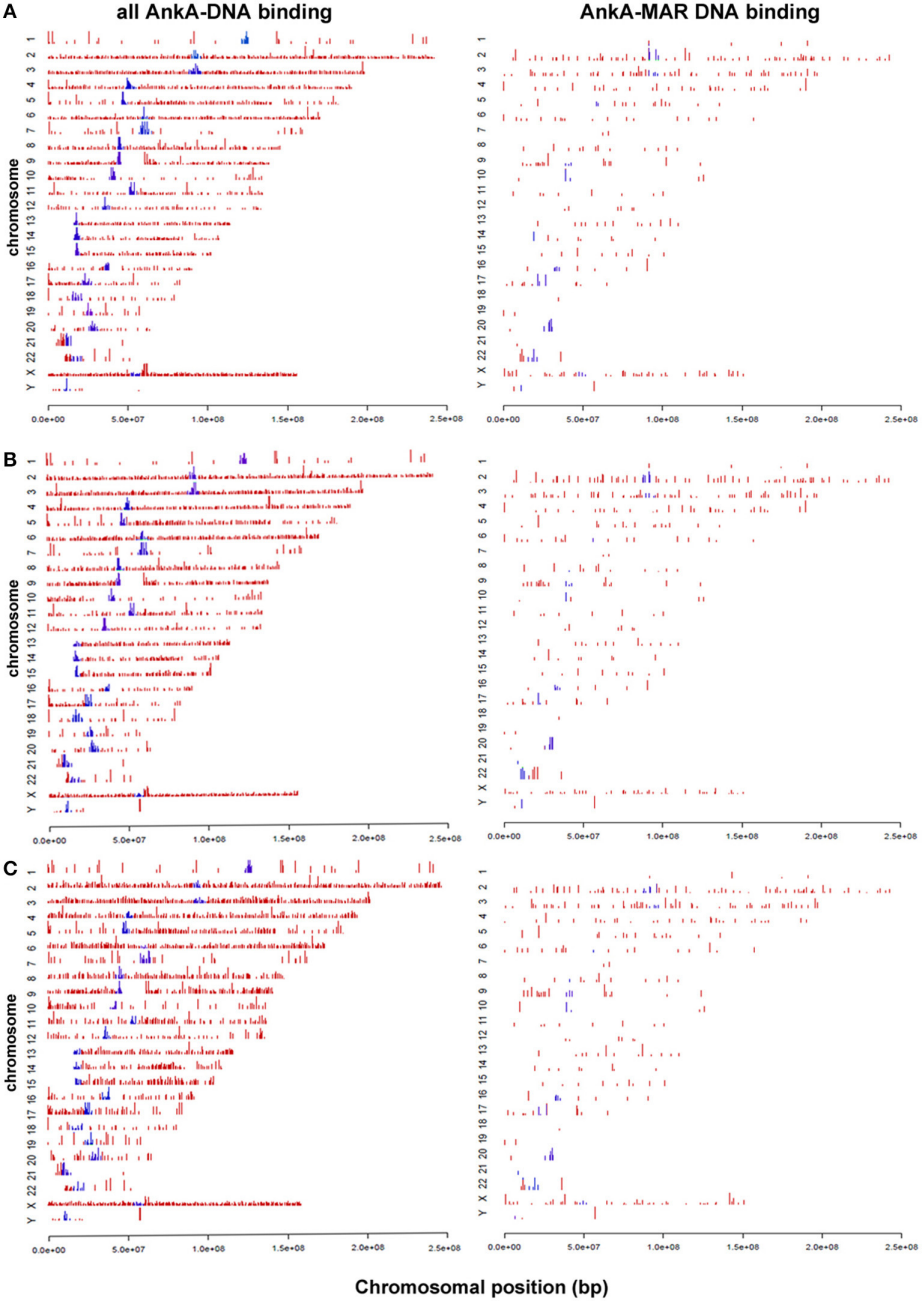
**AnkA binding sites across all chromosomes for each of 3 human neutrophil genomic DNA preparations (A–C)**. The **left** panels show the specific distribution and binding sites of AnkA to DNA. The **right** panels show the binding sites of AnkA to MARs identified across the genome using SMARTest (Genomatix) that predicts such regions based primarily on AT content. Blue bars represent AnkA binding at centromeres. Note the reproducible patterns and density of DNA binding and MAR binding by AnkA across all 3 neutrophil DNA preparations, and the marked enrichment at most centromeres.

Human peripheral blood neutrophils were isolated from 3 human subjects for DNA preparation. A total of 12,053 AnkA-bound regions were identified among the 3 neutrophil genomes, including 10,112 with unique sequence ranges whose average position differed from the next neighbor by >100 bp, and 4668 that are uniquely positioned near an annotated gene or gene feature (Figure 1). Among these, 1237 were shared in at least 2 genomes, and 324 AnkA binding sites shared the closest gene in all 3 genomes examined. Regions of AnkA binding to neutrophil DNA were mapped linearly along chromosomes, demonstrating that the changes in AnkA-DNA binding extended across all chromosomes (Figure 2) in a uniform pattern, and confirmed that AnkA-DNA binding occurred on a genome-wide scale. In fact, AnkA binding clusters were easily recognized since among 12,053 total binding sites across 3 genomes, 8650 (72%) binding sites occurred within 200,000 bp of the closest AnkA binding site and 4877 (40%) occurred within 12,500 bp of the closest AnkA binding site. Not annotated by CEAS was the observation of substantial AnkA binding at all centromeric regions (Figure 2) and to a lesser extent at telomeric regions, both included within distal intergenic domain classifications, and highly AT-enriched, a feature established for AnkA binding and important for MARs.

### Characterization of AnkA-DNA binding across common gene features

**Figure 3:**
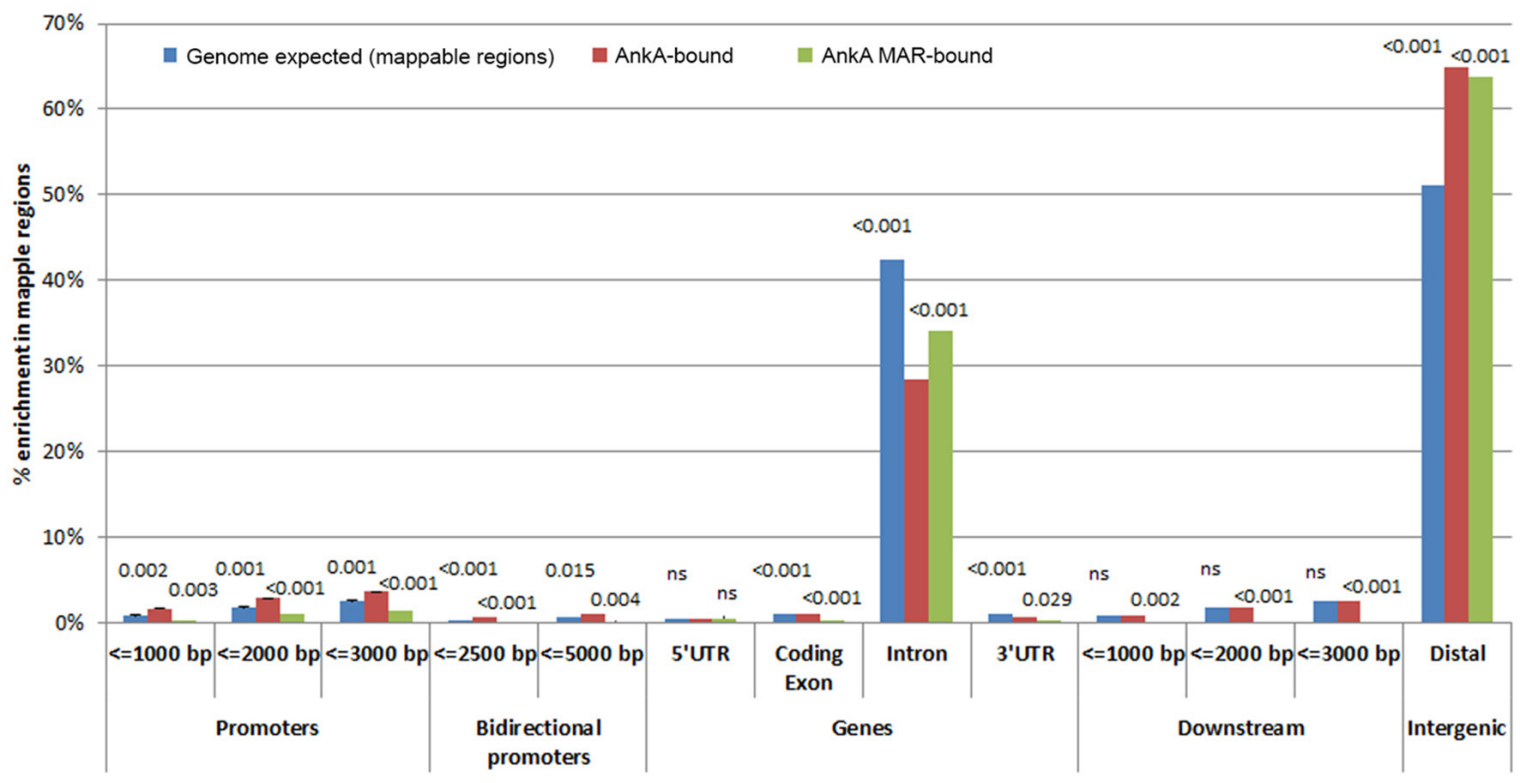
**Relative AnkA enrichment compared to mappable regions in the chromosomes [genome background (blue)] for all AnkA-enriched sites (red) and AnkA-MAR-enriched sites (green)**. Promoter regions are shown as distance upstream of transcriptional start sites, genes are shown as 5′ untranslated regions (UTR), coding exons, introns, and 3′UTRs, downstream regions are shown as the distance from the transcription termination sites, and intergenic regions using CEAS. The height of the bars represents the average of 3 separate experiments using individual donor neutrophils, including error bars. Numbers above the bars represent *p*-values of the comparisons for genomic background vs. enrichment at all AnkA-binding sites (left number) and vs. enrichment of AnkA-bound MARs (right number); *p* < 0.0168 were considered significant. Compared to genomic background, AnkA is most significantly enriched in distal intergenic regions, including when bound to MARs. Similarly, but to a much lower degree, AnkA is more significantly bound to the promoter regions ≤ 3000 bp upstream of TSSs, but this does not appear to be associated with specific MARs predicted in these promoters. ns, not significant.

AnkA is known to bind in *cis* promoter positions, where it impacts transcriptional activity by recruiting histone modifying proteins to modify chromatin structure and accessibility of transcription factors and/or RNA polymerase (Garcia-Garcia et al., 2009b; Rennoll-Bankert and Dumler, 2012; Rennoll-Bankert et al., 2015). Thus, we next sought to determine the gene features most prominently associated with AnkA binding throughout the genome. Average binding signals surrounding all transcription start sites (TSS), termination sites (TTS) and across genes for each individual donor and are shown in Figure 3. Across 3 neutrophil DNA profiles, AnkA binding showed similar relative profiles across gene features compared to mappable chromosomal regions. Chromosomes with significantly more AnkA enrichment compared to total mappable regions were identified on chromosomes 2, 3, 9, 13, 14, 17, 19, 22, and X. We first investigated whether AnkA localized to specific chromosomal features, and determined in all 3 genomes that binding was highly associated with distal intergenic regions, where ChIP regions that do not belong in any of other genomic features clustered with a mean of 64.9 vs. 51.2% among mappable regions. Over 62 and 50% of AnkA-binding sites mapped more than 3 or 20 kb from annotated genes, respectively. The median distance between individual AnkA binding sites and the closest gene was 21,037 bp (IQR 0-500,577).

**Figure 4:**
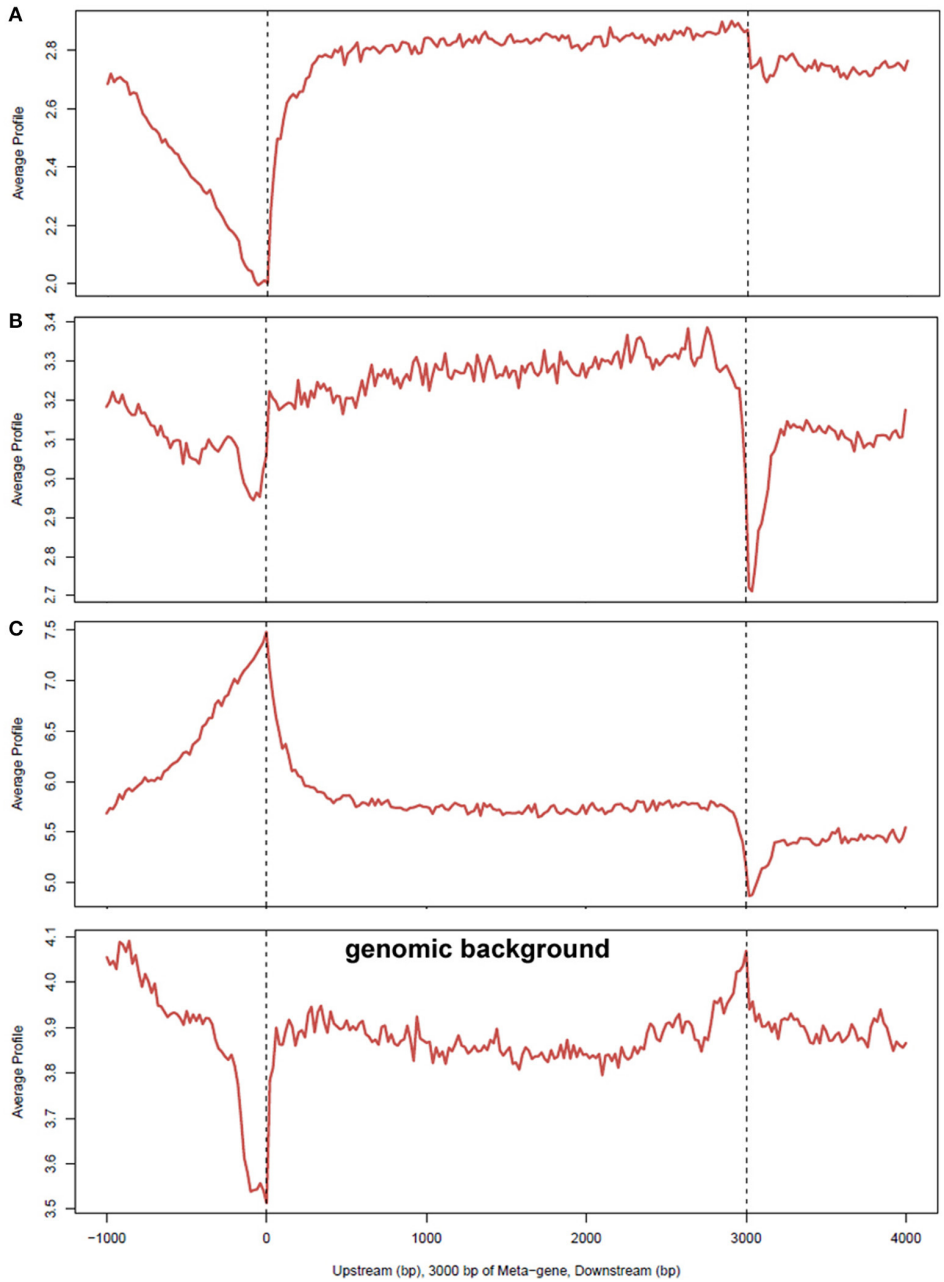
**Average profiles of AnkA binding enrichment over gene bodies normalized to 3000 bp, including upstream and downstream region**. The average signal profiles for AnkA binding at the upstream (left), gene body, and downstream (right) regions of genes, normalized to a 3000 bp gene is shown; **(A–C)** represent the data from the 3 individual neutrophil DNA samples used; genomic background is shown to demonstrate relative enrichment compared to mappable regions in the data sets. A reduction in AnkA enrichment was observed in 2 of 3 samples at and prior to the TSS and upstream. One sample showed a markedly different profile in this region, but all 3 samples were enriched in this region compared to genomic background. This patterns suggests a variable preference for AnkA enrichment at TSSs, but at lower levels than in adjacent gene bodies, or upstream or downstream regions. While all samples had a relatively level enrichment throughout the gene body, enrichment dropped at the TTS compared to the genomic background indicating that AnkA is largely excluded from these regions. AnkA enrichment returned to slightly lower levels than in gene bodies further downstream.

Although, AnkA binding in gene bodies comprised a minority of binding sites across genomic DNA, the proportion of binding across promoter regions < 3000 bp upstream of the TSS, and in bidirectional promoters < 5000 bp from the TSS was higher than expected. In contrast, AnkA binding was lower than expected in gene bodies, including the 5′ untranslated regions (UTR), coding exons, introns, 3′UTR, and regions < 3000 bp downstream of the TTS. Compared to surrounding gene features, 2 of the 3 samples showed declining AnkA binding at or in the surrounding ±1000 bp upstream of the TSS. AnkA binding sharply increased after the TSS and remained steady throughout the gene body until the TTS after which binding declined immediately downstream (Figure 4). One of the three samples (Figure 4C) had low AnkA binding in the regions 1000–2000 bps upstream of the TSS (where peak AnkA-binding was observed), that diminished to a steady level throughout the average gene body, and reached a nadir at the TSS. For all samples, binding at coding exons was not enriched, and although a high degree of binding was observed in introns (mean 27.1%), it was significantly less than the proportion of mappable intronic regions (mean 65.7%).

### AnkA binding and matrix attachment regions

AnkA has features of a MAR-binding protein, including binding to ATC single strand stretches where binding can be abrogated by mutating specific nucleotides within that ATC stretch. Additionally, there is a nuclear distribution that interacts with heterochromatin and forms a network around condensing chromatin (Caturegli et al., 2000; Garcia-Garcia et al., 2009b; Rennoll-Bankert et al., 2015). To discern whether genome-wide binding by AnkA is related to MAR binding, including potential binding *in vivo*, AnkA binding sites for each neutrophil DNA preparation were examined for MARs using SMARTest (Genomatix), and re-examined by CEAS as for direct AnkA-DNA binding. While AnkA MAR-binding sites are a subset of the overall AnkA binding sites, a total of 636 unique MAR binding sites among all three subjects' DNA interaction studies were identified. The distribution of binding (Figure 2, right panels) was very similar to that observed for all AnkA-DNA interactions: compared with genomic background, AnkA MAR-binding was highest in both intragenic regions and introns, but compared to total AnkA binding, binding to MARs was significantly lower in intragenic regions and significantly higher in introns. While statistically significant differences in total AnkA DNA-binding and MAR-binding were observed in gene-associated regions, the overall magnitude of differences was small, including at sites where AnkA binding to DNA was enriched (e.g., at promoter regions ≤ 3000 bp upstream of the TSS).

### AnkA binding and predicted interactions with nuclear lamina

**Figure 5:**
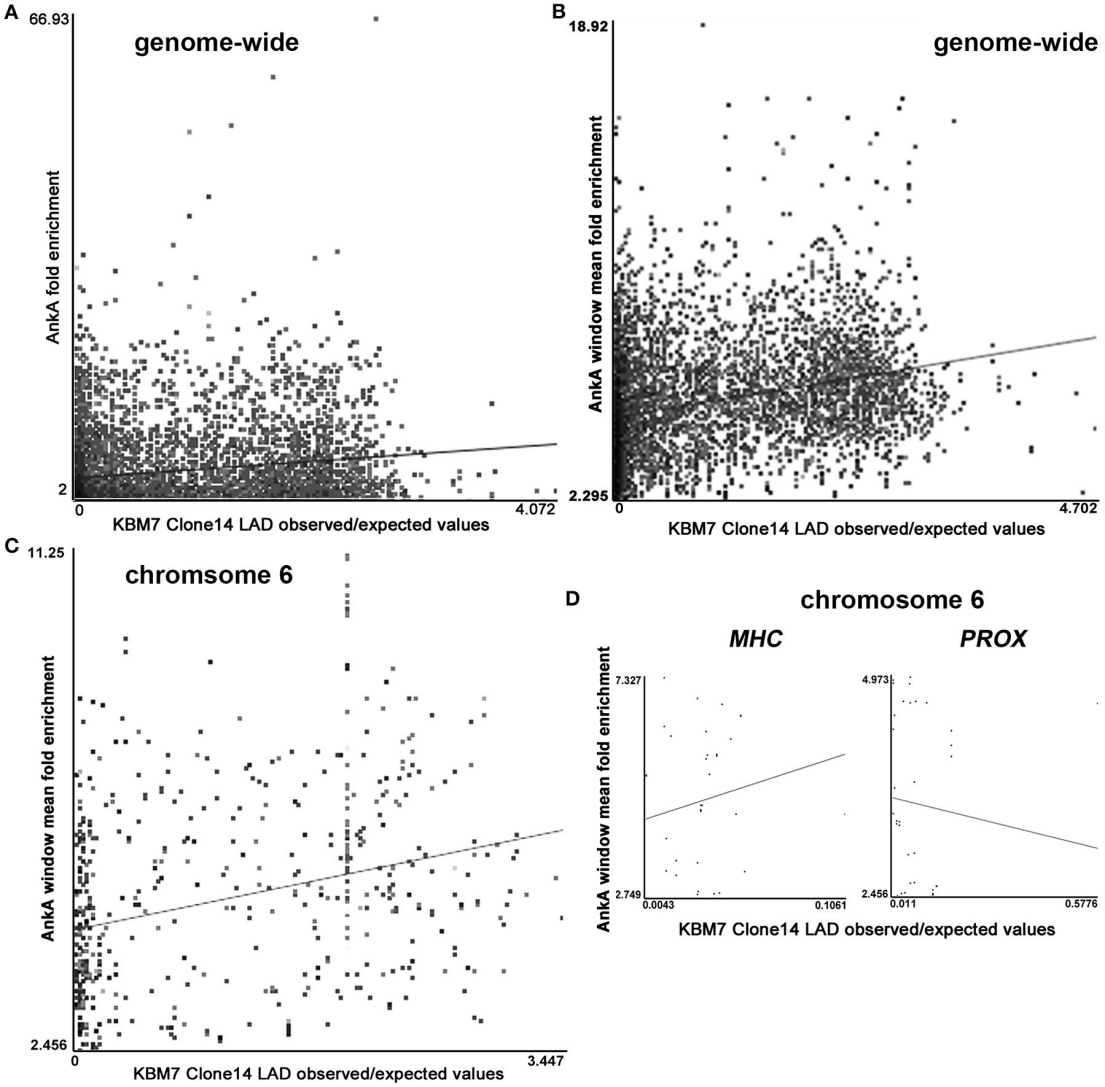
**Intersections of AnkA binding enrichment or window of AnkA binding enrichment with nuclear LADs (GEO accession GSE68263_Clone.14.1N.OE_LP150415). (A)** Shows the positive correlation across all chromosomes for individual AnkA binding site fold enrichment with LADs experimentally determined for haploid KBM-7 clone 14 chronic myelogenous leukemia cells. **(B)** Shows a stronger positive correlation when windows of AnkA binding average fold-enrichment over the entire genome are examined. **(C)** Strong positive correlation between AnkA windows that reflect average fold enrichment with LADs across chromosome 6. **(D)** AnkA window average fold enrichment and LADs correlation over the contiguous *MHC* locus (with marked upregulated gene expression) compared to the immediate downstream “*PROX*” region (lacking differential gene expression). Each dot represents the intersection values of KBM7 clone 14 cell average observed:expected (O:E) enrichment in the nuclear lamina vs. AnkA fold enrichment; the straight line represents the least squares fit for the data.

MARs and their binding partners are often found associated with the nuclear matrix and lamina as part of their genome organization and coordinate regulation functions (Rudd et al., 2004; Cai et al., 2006; Kohwi-Shigematsu et al., 2012; Pathak et al., 2014; Patrushev and Kovalenko, 2014; Yokota and Kanakura, 2014; Miyaji et al., 2015). There was a significant (*p* < 0.0021) genome-wide association between intersections of individual AnkA fold-enriched binding sites and haploid KBM-7 clone 14 lamina-associated domains (LADs; 6528 sites; *p* < 0.001; Figure 5A), and at individual chromosomes 1–4, 6, 9, 11, 13, 14, and X (Supplementary Table 2A and Figure S2A). The importance of these haploid myeloid cell clones allows an examination of lamina interactions, since only single chromosomes can establish these contacts, reducing an important potential confounding factor that occurs in diploid cells. Similarly, when adjacent AnkA binding sites are averaged over windows to discern long-range topologically-associated chromatin associations, there were significant associations between window average fold enrichment and KBM-7 LADs genome-wide (6348 sites; *p* < 0.001; Figure 5B), including significant associations at chromosomes 2–4, 6 (Figure 5C), 9, 11, 13, 14, 19, 22, and X (Supplementary Table 2B and Figure S2B). When the chromosome 6 *MHC* locus was examined, the positive relationship was similar, but not significant, probably owing to the small number of AnkA enriched windows in this short locus (34 sites; *r* = 0.133; *p* = 0.455; Figure 5D *MHC*). In contrast, examination of the downstream region of similar size, *PROX*, yielded a negative correlation (37 sites; *r* = −0.079; Figure 5D *PROX*), but no significant association either (*p* = 0.642).

### AnkA binding sites and noncoding RNA

**Figure 6:**
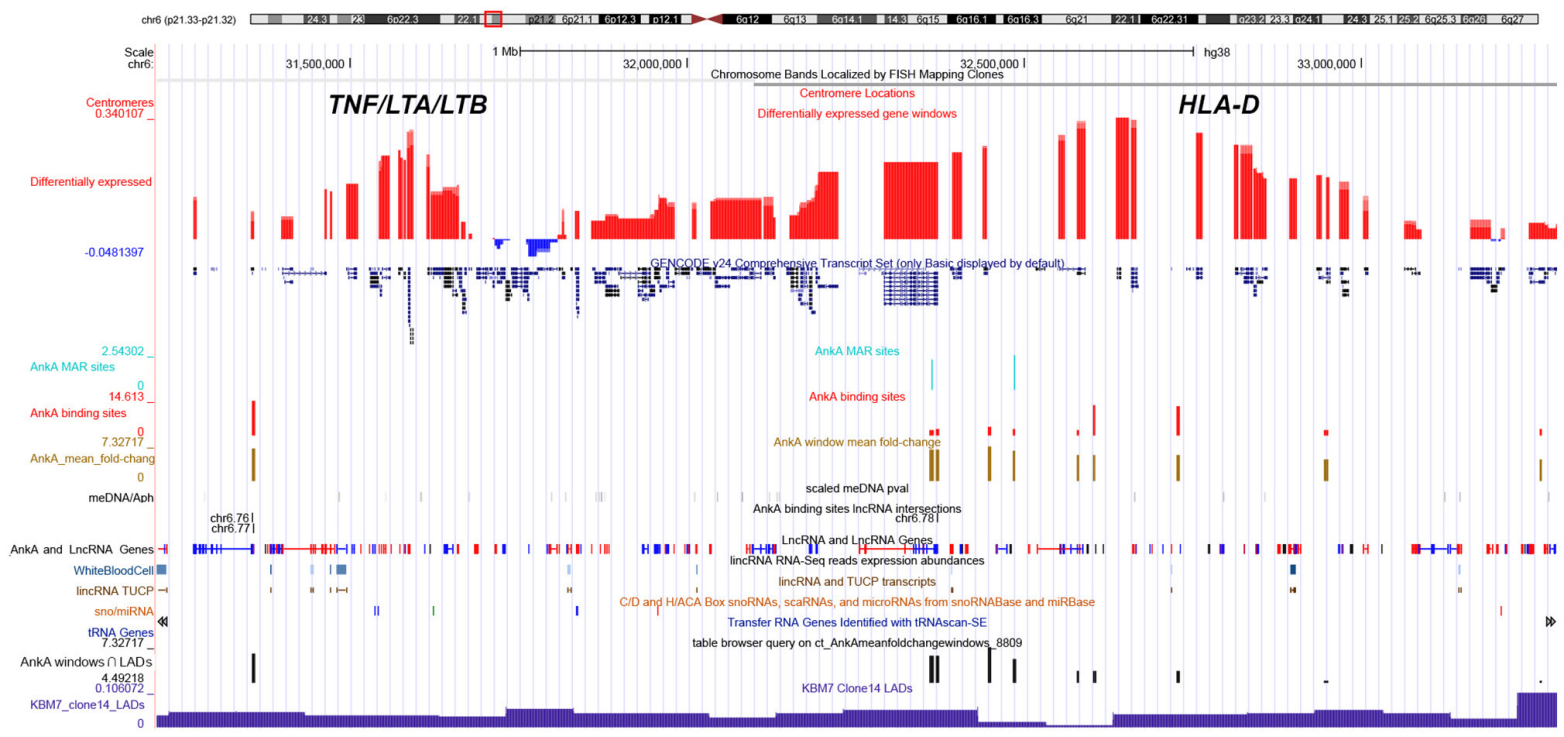
**UCSC genome browser comparison of genomic features and their relationship with AnkA binding at the *MHC* locus of chromosome 6**. The top line shows the ideogram for chromosome 6 with the *MHC* locus identified in the red box, and the specific location is delineated by bp from the telomeric p-arm of the chromosome followed by the chromosome band and centromere location. The large red and blue bars demonstrate the average differential transcription over approximately 10 Mbp windows in human neutrophils infected *ex vivo* by *A. phagocytophilum* for 24 h, and the GENCODE v22 gene locations are shown below. Custom features are as follows from top to bottom: light blue, AnkA MAR-binding sites (height is proportional to fold change); red, AnkA binding sites converged from all three samples sets (height is proportional to fold change); gold, mean AnkA fold change over ~50 Mbp windows, centered on the midpoint of the window (height is proportional to the average fold change centered at that window); black, unique DNA methylation marks with *A. phagocytophilum* infection of *ex vivo* human neutrophils at 24 h (intensity is proportional to *p*-values of methylated DNA at the site); AnkA binding sites lincRNA intersections black bands—represents the intersection of AnkA binding sites with the NONCODE positions for lincRNAs and lincRNA genes; LncRNA and LncRNA Genes blue and red bands—positions and direction (red forward, blue reverse) from NONCODE; lincRNA RNA-seq reads expression abundances light blue bands—lincRNA RNA-Seq reads expression abundances for human white blood cells; lincRNA TUCP, sno/miRNA, and tRNA Genes—tracks that show locations and directions for several classes of RNA sequences that do not code for a protein; mean AnkA fold change over ~50 Mbp windows intersected with KBM7 Clone 14 LADs black bars—height of bars shows relative enrichment of AnkA at nuclear lamina; KBM7 Clone 14 LADs dark blue histogram bars—shows regions often associated with three dimensional chromatin structure that interact with the nuclear lamina and are often inactive when in this conformation. AnkA data is cumulative of all 3 donor neutrophil DNA-AnkA interactions.

AnkA binding was greatest in intergenic regions, for which transcription is increasingly recognized and potentially functional. To determine whether AnkA binding co-localizes with established long intergenic non-coding RNAs (lincRNAs), we intersected the map of total AnkA-DNA binding with that of lincRNAs and their genes. The updated database in NONCODE (NONCODE2016_human.lncAndGene) possesses 231,415 entries, and the intersection with unique AnkA-binding sites identified 1574 unique intersections over all chromosomes (Figure 6). Of lincRNAs and lincRNA genes, 136,553 (59.0% [58.8–59.2% 95% CI]) are not directly associated with characterized genes annotated in GENCODE v22. In contrast, 1006 of 1574 (63.9% [61.5–66.3% 95% CI]), a significantly higher proportion of AnkA-bound lincRNA sites are not associated with genes, suggesting an increased association with lincRNA genes in intergenic regions. In contrast, only a small number (12–37) of miRNA and tRNA gene locations intersected with either specific AnkA binding sites or average windows of AnkA binding.

### AnkA binding and transcription

**Figure 7:**
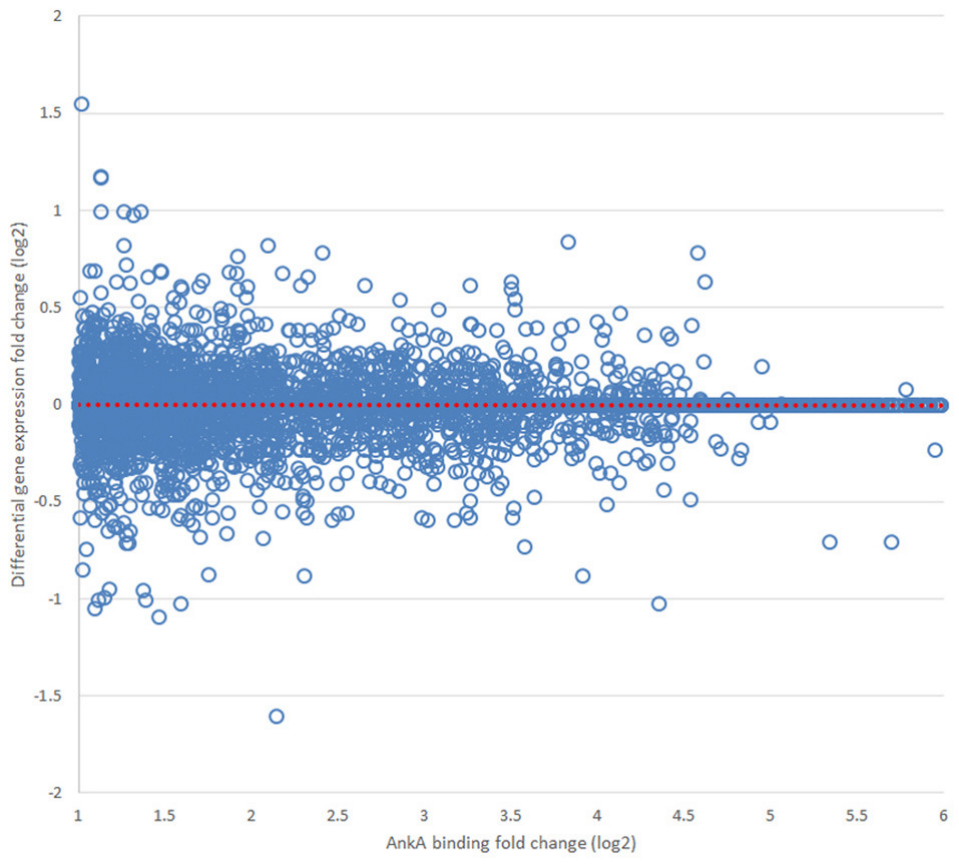
**Relationship of AnkA binding to average differential gene transcription in 10 Mbp windows over the entire genome**. Across all chromosomes, no relationship between AnkA binding fold change and differential transcription could be discerned. Data from all 3 donors is shown. The dotted red line is the least squares fitted regression line for the distribution.

**Figure 8:**
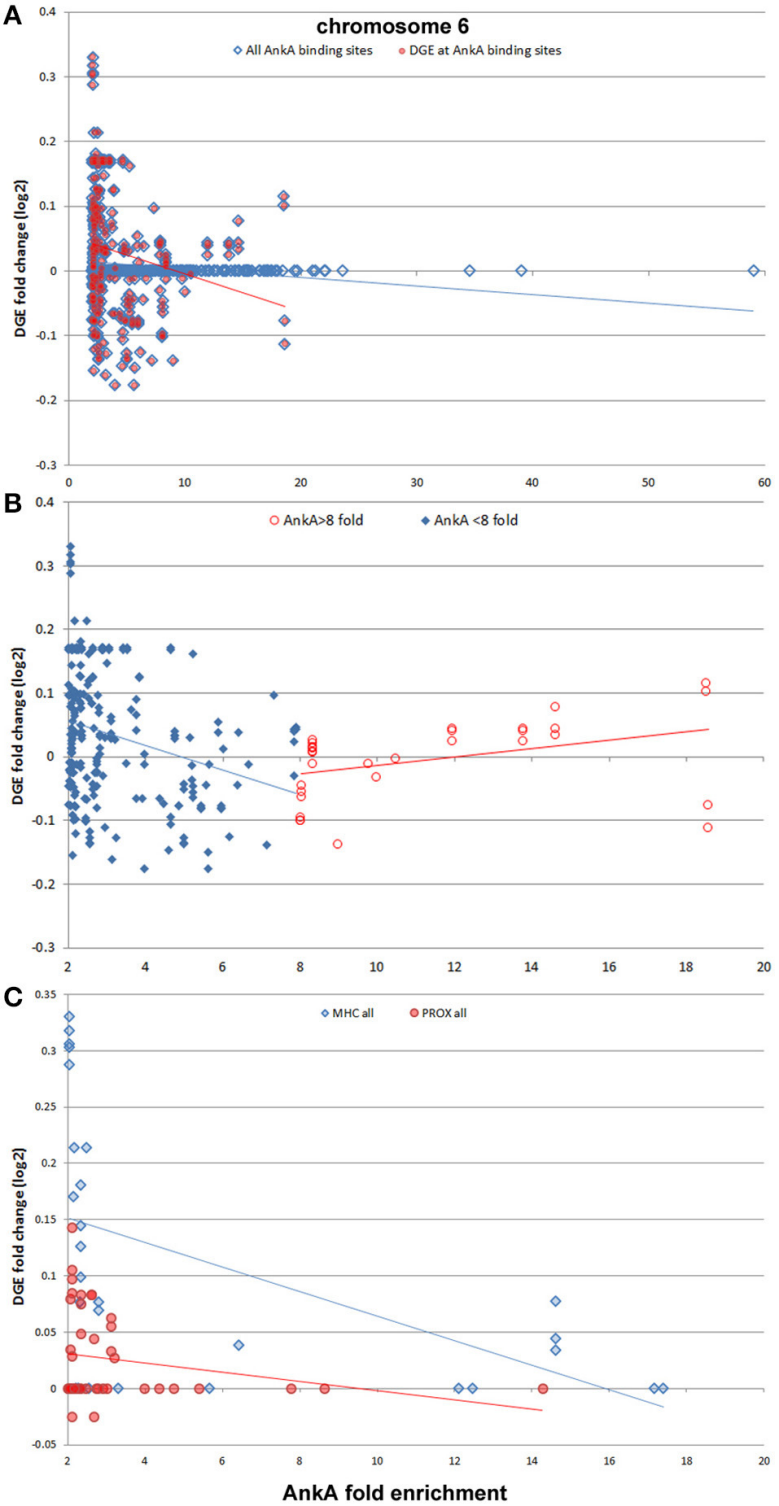
**AnkA binding and differential gene transcription at chromosome 6**. Across chromosome 6, including the known upregulated *MHC* locus, no specific correlation between AnkA binding and windows of gene transcription is identified (**A**, blue diamonds, blue regression line); however, when AnkA bound sites are examined for differential gene transcription, there is a significant overall transcriptional repression (red circles, red regression line). Despite the negative slope of the regression line, note the generally high differential transcription in windows where AnkA is bound with high fold enrichment. When this was examined separately **(B)** dividing into AnkA enrichment < 8-fold (blue diamonds, blue regression line) and ≥8-fold (red circles, red regression line), two distinct regression lines emerge, each significantly associated with (i) dampened transcription for windows with low AnkA binding, and (ii) increasing transcription for windows with higher fold levels of AnkA binding. The *MHC* locus is the most differentially regulated region on chromosome 6, with many genes and spanning windows demonstrating upregulated transcriptional activity. **(C)** The *MHC* region is examined by AnkA binding vs. differential gene expression window averages compared with the same features for the immediate downstream region (*PROX*). Here, AnkA has no observed effect on differential gene transcription at *PROX* (red circles, red regression line), but an overall downregulation at the *MHC* locus (blue diamonds, blue regression line) is observed with increasing AnkA binding, despite a similar increase in transcription with the highest levels of AnkA fold binding for which insufficient data points are available to demonstrate upregulation as observed at the chromosome level. Averaged data from all 3 donors is shown.

Finally, we sought to determine whether AnkA bound to DNA would be spatially organized in a manner to influence transcription of differentially expressed genes that are physically clustered on a chromosome. To determine the impact of AnkA binding in or around a gene, the relationship between AnkA fold increase vs. fold change in gene expression was investigated. Differential expression data obtained after infection of human neutrophils *ex vivo* by *A. phagocytophilum* (Borjesson et al., 2005; Sinclair et al., 2015) was intersected with all genes associated with AnkA binding. Overall, the genome-wide impact of AnkA binding on differential gene expression was minimal with −0.00073 mean fold-change and Pearson *R*^2^ 0.00016 (Figure 7). We previously examined the *MHC* locus, including the *TNF/LT* locus on chromosome 6, at which gene expression is broadly upregulated after DNA methylation induced by *A. phagocytophilum* infection (Sinclair et al., 2015). Over chromosome 6, there were 586 unique AnkA binding sites to 605 targeted genes among the 3 genomes examined; the UCSC Genome Browser view of this locus and associated genomic features is shown in Figure 8. Here, simple correlation between AnkA binding fold change and differential gene expression fold change included 44 unique sites on chromosome 6 but demonstrated no impact of AnkA binding (Pearson *R*^2^ 0.001). However, when genes without AnkA binding were excluded from the analysis, AnkA enrichment was correlated with reduced overall differential gene expression chromosome-wide (Pearson *R* = −0.184; *p* < 0.003). Closer examination suggested a bimodal distribution that was resolved into distinct differences for the influence of AnkA enrichment at or near differentially expressed genes across chromosome 6. For those genes with < 8-fold AnkA binding change, differential transcription fold change decreased (*R* = −0.285; *p* < 0.001), whereas when >8 AnkA fold change binding was examined, there was a strong increase in differential expression (*R* = 0.507; *p* < 0.005). When the *MHC* locus and the immediate downstream region were separately evaluated, the downstream *PROX* region had no significant change in differential gene expression compared with AnkA enrichment (*R* = −0.226; *p* > 0.05), but the *MHC* region retained the relationship between AnkA binding at low level enrichment and decreased gene expression (*R* = −0.517; *p* < 0.005). Although an apparent upregulation of gene expression was noted with higher AnkA enrichment at differentially expressed genes in the *MHC* locus, too few were available for statistical analysis. A similar analysis on chromosome 17 at the downregulated *MPO/LPO/EPX* locus revealed 223 unique AnkA enriched sites of 415 hits in the 3 DNA preparations, but no intersections at or within 3000 bp of genes (data not shown).

## Discussion

Prokaryotes evolved arrays of mechanisms and fitness attributes conducive for replication, spread, and survival (Bhavsar et al., 2007; Alix et al., 2011). For symbionts and mutualists, the outcomes are beneficial or neutral, whereas the outcomes for parasitic relationships are damaging and result in disease. While many disease-causing prokaryotes survive in an extracellular compartment, intracellular bacteria must both enter host cells undetected and alter the cellular machinery to survive, replicate, and spread. For obligate intracellular bacteria, host cell metabolism is shared with and required by the prokaryotic parasite or symbiont (Feldhaar and Gross, 2009; Omsland et al., 2013). Traditionally, bacterial pathogenesis studies focus on interactions of prokaryotic components with host cell surfaces, intracellular trafficking machinery, or cellular processes such signaling pathways (Ribet and Cossart, 2010). However, survival in intimate host cell niches depends on multiple alterations in host cell function, and these changes increasingly reflect the ability of the microbe to alter host cell gene expression (Hamon and Cossart, 2008; Bierne and Cossart, 2012; Rennoll-Bankert and Dumler, 2012; Silmon de Monerri and Kim, 2014).

It is now well recognized that bacterial pathogens can reprogram host gene expression by directly or indirectly altering accessibility of gene promoters via epigenetic modifications (Arbibe et al., 2007; Daniel et al., 2009; Garcia-Garcia et al., 2009b; Pennini et al., 2010; Rennoll-Bankert and Dumler, 2012; Lebreton et al., 2014; Silmon de Monerri and Kim, 2014). It is not surprising that transcriptional programs are altered with infection since recognition of an infectious agent's presence should initiate host responses (Akira and Takeda, 2004; Rogatsky and Adelman, 2014). However, the proof that microbial effectors directly target and alter these programs at the level of transcriptional regulation is an entirely new discovery (Bierne and Cossart, 2012; Silmon de Monerri and Kim, 2014). The impact of these observations goes to the conceptual problem of how microbes with limited genetic reservoirs manipulate broad functions encoded within genomes that average 1000 times larger. Clearly, individual protein effectors that target essential signaling pathways have an important effect, but often changes with infection are considerably larger and not well explained by targeting single pathways or as a cumulative result with arrays of effectors with similarly narrow targets. Given recent advances in the understanding of the regulation of eukaryotic gene expression by epigenetic mechanisms (Cheng and Blumenthal, 2010; Chou et al., 2011; Wu et al., 2012; Nora et al., 2013), including reprogramming of somatic cells and their differentiation into other cells with vastly different functions (Choi et al., 2009; Adams et al., 2013), it is apparent that microbes are likely to have evolved mechanisms to exert this control too.

*A. phagocytophilum* AnkA was described as the first secreted rickettsial effector in 2000 where it was found localized to heterochromatin of infected human granulocytes (Caturegli et al., 2000). It was characterized to bind host DNA and nuclear proteins in 2004 (Park et al., 2004), and in 2009 it was shown to be the factor responsible for silencing granulocyte respiratory burst (Garcia-Garcia et al., 2009b) by recruiting histone deacetylase 1 (HDAC1) to alter granulocyte function with infection (Garcia-Garcia et al., 2009a; Rennoll-Bankert et al., 2015). While some study has focused on control of eukaryotic gene expression by prokaryotic nucleomodulins (Bierne and Cossart, 2012; Rennoll-Bankert and Dumler, 2012; Silmon de Monerri and Kim, 2014), only AnkA of *A. phagocytophilum* has so far been demonstrated to exert its effects by direct DNA binding and modulation of chromatin structure to diminish *cis*-expression at specific genes. Given its genome-wide distribution, AnkA likely exerts influence over broad regions of multiple chromosomes (Park et al., 2004; Garcia-Garcia et al., 2009a,b; Rennoll-Bankert and Dumler, 2012). In fact, AnkA binds to specific regions of the *CYBB* promoter with attributes of eukaryotic MARs, and possesses biologic features of MARs including binding not to signature sequences, but to structure determined by stretches of ATC on single DNA strands; when the ATC structure is mutated, binding is abrogated or inhibited (Rennoll-Bankert et al., 2015). Moreover, many of the AT-enriched sites of AnkA attachment are predicted by bioinformatics to be MARs. Among epigenetic features that regulate gene expression, MARs are considered among the most potent, in part because of their ability to alter the topology of chromatin domains over long expanses, bringing together transcription factories or repressing genes configured to work coordinately (Cai et al., 2003, 2006; Kohwi-Shigematsu et al., 2012; Yokota and Kanakura, 2014).

MAR-binding proteins are considered “genome organizers” because of their ability to change the three-dimensional organization of chromatin itself at intra- and interchromosomal interactions to yield higher-order chromatin organization—important influences over the accessibility of DNA to gene regulatory components and the way genetic information is used (Kohwi-Shigematsu et al., 2012). It is estimated that the human genome possesses over 60,000 MARs, but only a fraction has been functionally characterized (Rudd et al., 2004; Pascuzzi et al., 2014; Pathak et al., 2014). While their precise distribution in the human genome is not well established, they are often associated with regions that regulate *cis* transcription, such as at origins of replication, TSS, and RNA Polymerase II sites and exons of highly transcribed genes (Pascuzzi et al., 2014; Pathak et al., 2014). MARs are also highly represented among non-coding DNA, where they serve as tether sites for proteins that bring distant co-regulated sites together in chromatin loops extending from the nuclear matrix (Kisseljova et al., 2014; Patrushev and Kovalenko, 2014; Pathak et al., 2014; Kind et al., 2015). Such sites are often located in long AT-rich intergenic regions separated by as much as 200 kb (Miyaji et al., 2015). Other potentially important epigenetic factors include DNA methylation, regulated miRNA and lincRNA, among other possibilities. We previously showed a marked increase in DNA methylation with *A. phagocytophilum* infection and its linkage to differential gene expression (Sinclair et al., 2015). Here, we investigated the genome-wide distribution of AnkA binding to discern patterns that might predict how it functions as an epigenetic modifier of transcriptional reprogramming.

The predictions that surface from such information suggest that AnkA could bind in multiple locations and serve several functions: (1) *cis*-acting—such as direct regulation at or upstream of gene promoters, and (2) *trans*-acting—by forming tethering points for DNA loops to promote transcription factories or to silence transcription by sequestering critical regulatory regions into the heterochromatic nuclear matrix (Kisseljova et al., 2014; Kind et al., 2015). The resulting genome-wide mapping of AnkA binding and enrichment at specific genomic positions supports both of these hypotheses in that the majority of binding sites are enriched within distal intergenic regions and to a lesser extent in the proximal promoters within 3000 bp of gene TSSs.

While the distal intergenic regions comprise the majority of DNA in the genome (Patrushev and Kovalenko, 2014), that it is so substantially enriched in AnkA binding, and reproducibly so over 3 distinct human genomic DNA preparations, strongly supports these regions as the major binding sites and likely the regions for greatest impact over transcriptional reprogramming. That AnkA binding has little overall effect on individual gene transcription is illustrated by the lack of correlation between differential gene expression when intersected with specific AnkA-binding locations. However, if AnkA binding at specific genes exerts a greater influence as a result of chromatin organization changes, a direct AnkA-binding site to gene intersection might not reflect this relationship if each does not precisely overlap, as expected for MARs and genome organizers. To understand this possible relationship better, we examined average differential gene transcription over genomic windows on chromosome 6 (window median length 1.63 Mb, IQR 0.42–3.42 Mb; median 10 genes, IQR 8–12 genes). These locations and differential expression fold changes were intersected with specific AnkA enrichment sites and revealed significant negative correlation (*r* = −0.18; *p* = 0.003). However, these data also revealed a bimodal curve of gene windows with upregulated transcription highly correlated with high AnkA fold-enrichment. When the bimodal distribution was examined as AnkA enrichment < 8- or ≥8-fold, we identified concurrent significant correlation of downregulated windows with low AnkA fold-enrichment and significant correlation of upregulated windows with higher AnkA fold-enrichment. The specific reasons for this dichotomy are not well explained by the data, and need further investigation to determine if these findings are the result of how AnkA tethers DNA regions to into various loop configurations to either restrict or promote transcriptional activity.

To examine this concept further, we sought to identify a relationship between AnkA binding and the nuclear lamina where MARs and genome organizers tether DNA to change three-dimensional structure and looping, and bring long stretches of DNA with functionally-related genes in proximity of RNA polymerase and other regulatory molecules. Here, we selected to examine the distribution of specific chromatin domains identified as interacting with Lamin B1 in 118 clones of the haploid chronic myelogenous leukemia cell line KBM-7 as a surrogate for human neutrophils (Kind et al., 2015). The haploid nature of these clones provides an opportunity to examine such interactions since only a single chromosome can establish contacts, reducing an important potential confounding factor. Remarkably, AnkA individual site enrichment or window average AnkA enrichment were significantly correlated with LADs genome-wide. Significant correlation of AnkA enrichment at the nuclear lamina was predicted across multiple but not all chromosomes (excluding chr8 that was diploid and chrY that was not present), suggesting that the process was not random. This association extended to chromosome 6, where large scale differential gene expression is well documented with *A. phagocytophilum* infection, and these data suggest further that this process could be relevant to the *MHC* locus.

Once thought to be “junk DNA,” the intergenic noncoding regions account for transcription of 85% of cellular RNA vs. 3% for protein-encoding genes (Elgar and Vavouri, 2008; Pennisi, 2012). It has become clear that noncoding RNAs, such as lincRNAs, miRNAs, tRNAs among others likely play major roles in gene regulation (Chavali et al., 2011; Ching et al., 2015; Rodkvamtook et al., 2015; Eidem et al., 2016; Nam et al., 2016). That the majority of AnkA binding sites localize to such regions suggests the need to investigate their potential interactions with ncRNAs. While co-localization in map intersections show that AnkA binding is modestly enriched in lincRNAs sites not associated with protein-encoding genes, only 12–37 genomic sites are identified for AnkA binding and miRNAs and tRNAs (sno/miRNA, miRNA 21, and tRNA genes). The relatively few intersections compared to the total repertoire of lincRNA genes does not provide strong support for a major role of AnkA in regulating these loci.

These data further raise the question of whether AnkA with known MAR-binding properties exert influence by organizing chromatin from these locations. While the majority of AnkA binding sites were not also predicted to overlap MARs, the prediction tool uses weight matrices that are associated with the AT-rich class of MARs, can have false positives, or may not identify MARs with other features (www.genomatix.de/online_help/help_gems/SMARTest.html). That this is likely stems from an analysis of chromosome 6 MARs using the SMARTest where only 0.30% of the chromosome is predicted to contain a MAR. In contrast, studies in humans, *Drosophila* and *Arabidopsis* indicate that the overall content of MARs is likely more on the order of 2.5–7.7%, suggesting a significant underestimation by the bioinformatics approach (Frisch et al., 2002; Rudd et al., 2004; Shaposhnikov et al., 2007; Pascuzzi et al., 2014; Pathak et al., 2014). In fact, the SMARTest failed to identify the experimentally-corroborated AnkA-bound MAR in the *CYBB* promoter. Thus, whether MARs are involved in the binding of AnkA throughout intergenic regions will need to be determined experimentally.

AnkA is demonstrated to regulate *cis* gene expression at *CYBB* by binding to the proximal promoter, recruiting HDAC1 leading to deacetylation of histone H3 thereby excluding binding to RNA polymerase II in the nuclear matrix (Rennoll-Bankert et al., 2015). A similar pattern of histone H3 deacetylation and RNA polymerase II exclusion also occurs after *A. phagocytophilum* infection of granulocytes at a number of defense gene promoters, some clustered in specific chromosomal loci, suggesting that AnkA also regulates these genes (Garcia-Garcia et al., 2009a). Here, AnkA was significantly enriched in the 3000 bp upstream of TSSs, but not downstream of TTSs, suggesting a role in direct *cis* regulation. The explanation for the variability of AnkA enrichment at TSSs over the 3 genomes is unclear and needs to be further investigated, and studies that examine AnkA binding in infected cells could help to resolve the discrepancy. However, among the 3 genomes queried, AnkA was found at or within 3000 bp upstream or downstream of 360 genes, for which differential gene transcription data was available for only 184. Among this abridged set, there was no correlation between AnkA fold-enrichment and differential gene transcription.

While AnkA binding across chromosome 6 is associated with transcriptional upregulation when highly enriched, and with transcriptional downregulation with lower levels of enrichment, it is still unclear whether this applies to the highly regulated *MHC* locus. We previously demonstrated that *A. phagocytophilum*-induced DNA methylation is associated with transcriptional upregulation at the MHC locus on chromosome 6 (Sinclair et al., 2015). Here, most AnkA binding was not highly enriched and the overall relationship between AnkA binding and differential transcription at the *MHC* locus was downregulation; in this analysis only a few gene transcription windows were identified that were highly enriched for AnkA, confounding further statistical analysis. However, it is apparent from the Genome Browser view (Figure 6) that AnkA enrichment occurs at regular intervals across the *MHC* locus, including at the *TNF/LT* locus, suggesting a role as a genome organizer in those regions of significant upregulated expression.

Caveats of this study are that binding to DNA *in vitro* might not reflect AnkA distribution in an infected cell. Additionally, the intersections used to compute LAD and MAR binding are based on surrogate models and *in silico* predictions that might not reflect the situations within infected neutrophils or granulocytes. Still, AnkA binds broadly throughout the human genome in reproducible locations on each chromosome in 3 replicated DNA binding experiments. It binds to over 10,112 sites with unique sequence ranges and although binding varied, average enrichment was nearly seven-fold at individual sites. AnkA is enriched in (i) distal intergenic regions, (ii) in promoters—the 3000 bp regions upstream of TSSs, (iii) in the nuclear lamina, and is associated with differential gene transcription in regions of chromosome 6 and the *MHC* locus; binding to both centromeric and telomeric regions needs more investigation, although it is well established that these regions are highly AT-enriched—a characteristic of AnkA binding sites. Aside from the latter, these observations are consistent with the hypothesis that AnkA is multifunctional, acting as both a genome organizer and as a *cis*-regulator of gene transcription. The former functions could play an important role in the coordinated reprogramming of neutrophils with infection by *A. phagocytophilum*, or as observed with AnkA transfection alone (Garcia-Garcia et al., 2009a,b; Sinclair et al., 2014; Rennoll-Bankert et al., 2015). Considerable experimental study, including direct chromatin immunoprecipitation from transfected and *A. phagocytophilum*-infected cells will be required to confirm and extend the observations, while direct evaluation of long-range chromatin changes influenced by AnkA should be examined using modern chromatin conformation approaches. If the hypothesis is proven, the means by which *A. phagocytophilum* reprograms cells would be entirely novel in the realm of prokaryotic fitness adaptations and as a pathogenetic mechanism, and could shed light on how obligate intracellular microbes reprogram host cells in fitness and in disease.

## Author contributions

JSD conceived and directed the study, conducted analyses, and wrote the manuscript. SS conceived and initiated the study, and contributed intellectual content. VP conducted the biological experimentation and contributed intellectual content. AS conducted the bioinformatics analyses and contributed intellectual content.

## Funding

Funding to support this work was provided by National Institutes of Health (NIH)/National Institutes of Allergy and Infectious Diseases (NIAID) grant R01AI044102 to JSD, by funds supplied through the University of Maryland School of Medicine and grant PAT-74-2977 from the Uniformed Services University of the Health Sciences. The opinions expressed herein are those of the author(s) and are not necessarily representative of those of the Uniformed Services University of the Health Sciences (USUHS), the Department of Defense (DOD); or, the United States Army, Navy, or Air Force.

### Conflict of interest statement

The authors declare that the research was conducted in the absence of any commercial or financial relationships that could be construed as a potential conflict of interest.
