# Supplementary material for: Genome-Wide Anaplasma phagocytophilum AnkA-DNA Interactions Are Enriched in Intergenic Regions and Gene Promoters and Correlate with Infection-Induced Differential Gene Expression
Source: Front Cell Infect Microbiol. 2016 Sep 20;6:97. doi: 10.3389/fcimb.2016.00097 (PMC5028410; doi:10.3389/fcimb.2016.00097)
Supplement: Supplementary file 1 [file Table1.pdf]

Supplemental Table 1. Total reads, mapped regions, % mapped reads, and other statistics for the AnkA-DNA binding experiments.

Sample.ID	Total Reads	Mapped Reads	% Mapped Reads	Genome Covered Bases	Mapped Read Bases	Per Base Coverage
**A**	112,912,344	107,038,403	94.80	2,693,140,238	10,756,218,662	3.9939
**B**	111,274,016	99,797,540	89.69	2,445,365,145	10,036,541,225	4.1043
**C**	166,591,778	149,512,538	89.75	2,560,156,909	15,014,035,992	5.8645
