# Supplementary material for: Genome-Wide Anaplasma phagocytophilum AnkA-DNA Interactions Are Enriched in Intergenic Regions and Gene Promoters and Correlate with Infection-Induced Differential Gene Expression
Source: Front Cell Infect Microbiol. 2016 Sep 20;6:97. doi: 10.3389/fcimb.2016.00097 (PMC5028410; doi:10.3389/fcimb.2016.00097)
Supplement: Supplementary file 2 [file Table2.PDF]

Supplemental Table 2A. Correlation of AnkA enrichment binding sites with KMB-7 clone 14 average O/E displacement into nuclear lamina over all haploid chromosomes.

Position and # of data points in intersection	Correlation coefficient r	r2	Track	Minimum	Maximum	Mean	Variance	Standard deviation	Regression line y = m*x + b m	b	p value[1]
chr1:2,652,021-236,717,056	0.4602	0.2118	AnkA binding sites	2.21	33.2	4.113	13.76	3.709	3.789	3.225	< 0.00001
161,113 data points			KBM7_clone14_LADs	0.00995	2.15	0.2343	0.2029	0.4505			
chr2:155,226-242,046,295	0.151	0.02281	AnkA binding sites	2	66.9	5.249	21.47	4.634	0.7123	4.628	< 0.00001
1,981,512 data points			KBM7_clone14_LADs	0	3.39	0.8725	0.9655	0.9826			
chr3:90,557-198,141,856	0.1738	0.03022	AnkA binding sites	2	24.4	5.882	21.49	4.636	0.8334	5.009	< 0.00001
1,568,669 data points			KBM7_clone14_LADs	0	3.25	1.048	0.9352	0.9671			
chr4:206,414-190,073,254	0.1983	0.03931	AnkA binding sites	2	30.1	6.498	25.65	5.065	1.095	5.324	< 0.00001
885,330 data points			KBM7_clone14_LADs	0	2.94	1.072	0.8405	0.9168			
chr5:344,136-180,131,368	0.09822	0.009647	AnkA binding sites	2.01	32.8	7.188	30.4	5.514	0.5905	6.719	0.019238
459,696 data points			KBM7_clone14_LADs	0	3.03	0.7938	0.8409	0.917			
chr6:139,454-170,497,467	0.277	0.07671	AnkA binding sites	2	59	4.741	18.67	4.321	1.288	3.668	< 0.00001
1,095,676 data points			KBM7_clone14_LADs	0	3.45	0.8337	0.8634	0.9292			
chr7:904,629-158,916,149	0.1948	0.03795	AnkA binding sites	2.01	51.7	6.781	74.32	8.621	2.639	5.702	0.000055
119,373 data points			KBM7_clone14_LADs	0	2.38	0.4089	0.4049	0.6364			
chr9:142,869-138,187,779	0.2685	0.07207	AnkA binding sites	2.01	25.7	5.206	18.74	4.329	1.207	4.097	< 0.00001
909,898 data points			KBM7_clone14_LADs	0	4.57	0.9195	0.9272	0.9629			

chr10:56,764-133,741,659	0.2652	0.07033	AnkA binding sites	2.02	43	6.369	32	5.657	2.83	5.243	< 0.00001
249,959 data points			KBM7_clone14_LADs	0.0147	2.54	0.3977	0.2811	0.5302			
chr11:891,962-134,283,055	0.3699	0.1368	AnkA binding sites	2	52.3	4.983	35.04	5.919	2.652	3.68	< 0.00001
369,659 data points			KBM7_clone14_LADs	0	3.21	0.4914	0.6815	0.8255			
chr12:397,578-133,223,414	0.251	0.06299	AnkA binding sites	2.01	30.8	7.17	31.23	5.588	1.527	5.8	< 0.00001
189,265 data points			KBM7_clone14_LADs	0.0116	2.74	0.8972	0.8436	0.9185			
chr13:19,103,832-114,201,318	0.2801	0.07846	AnkA binding sites	2	23.2	5.523	23.27	4.824	1.58	4.384	< 0.00001
522,334 data points			KBM7_clone14_LADs	0.0149	3.25	0.7209	0.7314	0.8552			
chr14:18,894,295-106,753,954	0.3938	0.1551	AnkA binding sites	2	35.5	5.565	24.63	4.963	2.816	4.239	< 0.00001
575,881 data points			KBM7_clone14_LADs	0	2.96	0.4708	0.4815	0.6939			
chr15:19,899,996-101,764,988	-0.05189	0.002693	AnkA binding sites	2.03	26.4	5.892	25.42	5.041	- 0.2128	6.126	0.348481
232,054 data points			KBM7_clone14_LADs	0	4.7	1.101	1.512	1.229			
chr16:241,630-90,108,157	-0.04881	0.002382	AnkA binding sites	2	21.1	4.842	17.79	4.218	- 0.2279	4.98	0.43824
380,793 data points			KBM7_clone14_LADs	0	2.7	0.6048	0.8159	0.9033			
chr17:198,951-82,588,500	0.05403	0.00292	AnkA binding sites	2.01	26.9	6.134	24.12	4.911	0.7626	5.985	0.272403
371,660 data points			KBM7_clone14_LADs	0	2.01	0.1953	0.1211	0.3479			
chr18:104,679-79,100,213	-0.04251	0.001807	AnkA binding sites	2.18	28.1	10.14	50.55	7.11	- 0.3586	10.48	0.715032
63,228 data points			KBM7_clone14_LADs	0.0208	2.48	0.9413	0.7102	0.8428			
chr19:377,643-56,748,975	-0.123	0.01512	AnkA binding sites	2.03	17.1	3.804	6.294	2.509	-1.127	4.04	0.027077

234,243 data points			KBM7_clone14_LADs	0.0196	0.919	0.2095	0.07498	0.2738			
chr20:2,391,144-63,254,483	0.3718	0.1382	AnkA binding sites	2	21.4	5.846	23.42	4.839	3.195	4.266	< 0.00001
85,184 data points			KBM7_clone14_LADs	0.0113	1.9	0.4946	0.3173	0.5633			
chr21:8,611,169-14,183,275	0.3089	0.09539	AnkA binding sites	2.62	28.1	4.247	6.067	2.463	0.4928	3.218	0.000083
13,858 data points			KBM7_clone14_LADs	0	3.92	2.089	2.383	1.544			
chr22:15,622,801-29,122,188	0.2717	0.0738	AnkA binding sites	2.09	5.3	3.685	0.7954	0.8919	0.9534	3.4	0.000075
76,725 data points			KBM7_clone14_LADs	0	0.823	0.2988	0.06459	0.2541			
chrX:2,860,219-155,768,174	0.1818	0.03306	AnkA binding sites	2	25.8	6.236	19.15	4.377	0.8252	5.338	< 0.00001
1,937,895 data points			KBM7_clone14_LADs	0	4.48	1.088	0.9299	0.9643			
OVERALL	0.1948	0.03796	AnkA binding sites	2	66.9	5.681	23.08	4.805	0.9971	4.843	< 0.00001
12,484,005 data points			KBM7_clone14_LADs	0	4.7	0.8405	0.8814	0.9388			

[1] Bonferroni correction for α=0.05: 0.002173913

Supplemental Table 2B. Correlation of AnkA mean fold-change window (~53 Mbp) with KMB-7 clone 14 average O/E displacement into nuclear lamina over all haploid chromosomes.

Position and # of data points in intersection	Correlation coefficient r	r2	Track	Minimum	Maximum	Mean	Variance	Standard deviation	Regression line y = m*x + b m	b	P value[1]
chr1:2,652,021-176,752,365	0.2285	0.0522	AnkA_mean_fold-change_windows	2.78	11.1	5.681	5.058	2.249	1.314	5.431	< 0.00001
138,386 data points			KBM7_clone14_LADs	0.00995	2.1	0.19	0.1528	0.3909			
chr2:855,758-241,916,981	0.3638	0.1324	AnkA_mean_fold-change_windows	2.3	16.4	5.501	3.806	1.951	0.7199	4.871	< 0.00001
1,961,650 data points			KBM7_clone14_LADs	0	3.39	0.8755	0.9721	0.9859			
chr3:1,482,726-197,222,547	0.2503	0.06267	AnkA_mean_fold-change_windows	2.5	10.6	6.01	2.91	1.706	0.4434	5.547	< 0.00001
1,549,491 data points			KBM7_clone14_LADs	0	3.25	1.046	0.928	0.9633			
chr4:206,414-190,073,254	0.288	0.08297	AnkA_mean_fold-change_windows	2.74	10.3	6.2	3.165	1.779	0.5589	5.601	< 0.00001
885,330 data points			KBM7_clone14_LADs	0	2.94	1.072	0.8405	0.9168			
chr5:415,947-170,244,623	0.02443	0.000597	AnkA_mean_fold-change_windows	3.13	13.4	7.487	4.891	2.212	0.05859	7.439	0.56658
441,246 data points			KBM7_clone14_LADs	0	3.03	0.821	0.8503	0.9221			
chr6:289,964-168,842,754	0.2731	0.07457	AnkA_mean_fold-change_windows	2.46	11.3	5.026	3.068	1.752	0.5115	4.585	< 0.00001
1,056,857 data points			KBM7_clone14_LADs	0	3.45	0.8609	0.8743	0.935			
chr7:25,041,468-152,408,205	0.1302	0.01696	AnkA_mean_fold-change_windows	3.45	18.9	8.393	20.5	4.528	0.844	7.965	0.008381
90,329 data points			KBM7_clone14_LADs	0	2.38	0.5064	0.488	0.6985			
chr9:2,946,394-137,570,837	0.4415	0.1949	AnkA_mean_fold-change_windows	2.48	10.1	5.16	3.374	1.837	0.8435	4.378	< 0.00001
878,109 data points			KBM7_clone14_LADs	0	4.57	0.9273	0.9242	0.9614			
chr10:421,741-128,465,008	0.08773	0.007696	AnkA_mean_fold-change_windows	3.06	10.3	6.755	2.035	1.427	0.2278	6.658	0.102907

222,874 data points			KBM7_clone14_LADs	0.0147	2.54	0.4276	0.3018	0.5494			
chr11:1,435,409-127,369,344	0.3246	0.1054	AnkA_mean_fold-change_windows	2.43	11.4	5.073	6.503	2.55	0.9918	4.578	< 0.00001
336,966 data points			KBM7_clone14_LADs	0	3.21	0.499	0.6968	0.8348			
chr12:5,117,652-131,211,014	0.3155	0.09953	AnkA_mean_fold-change_windows	3.62	12.6	8.089	4.407	2.099	0.7199	7.389	< 0.00001
174,050 data points			KBM7_clone14_LADs	0.0237	2.74	0.9727	0.8464	0.92			
chr13:19,103,832-113,636,063	0.5613	0.315	AnkA_mean_fold-change_windows	2.52	9.16	5.342	2.353	1.534	1.003	4.603	< 0.00001
510,324 data points			KBM7_clone14_LADs	0.0149	3.25	0.7372	0.7371	0.8585			
chr14:18,894,295-105,618,466	0.53	0.2809	AnkA_mean_fold-change_windows	2.87	15	5.694	4.878	2.209	1.66	4.888	< 0.00001
551,995 data points			KBM7_clone14_LADs	0	2.96	0.4854	0.4971	0.7051			
chr15:19,899,996-100,803,676	-0.216	0.04665	AnkA_mean_fold-change_windows	3.37	11.9	6.298	3.349	1.83	-0.3177	6.663	0.000084
219,783 data points			KBM7_clone14_LADs	0	4.7	1.148	1.548	1.244			
chr16:2,449,463-90,108,157	-0.1816	0.03299	AnkA_mean_fold-change_windows	2.58	8.62	5.067	2.058	1.435	-0.2835	5.249	0.004164
358,293 data points			KBM7_clone14_LADs	0	2.7	0.641	0.8449	0.9192			
chr17:287,669-64,923,050	0.0426	0.001815	AnkA_mean_fold-change_windows	2.45	10.1	6.079	3.41	1.847	0.2183	6.033	0.394887
340,757 data points			KBM7_clone14_LADs	0	2.01	0.208	0.1299	0.3604			
chr18:12,979,376-68,786,913	0.06262	0.003921	AnkA_mean_fold-change_windows	5.8	11.3	8.549	2.208	1.486	0.1129	8.423	0.623135
38,119 data points			KBM7_clone14_LADs	0.0208	2.34	1.109	0.6797	0.8244			
chr19:8,740,781-50,125,075	0.5751	0.3307	AnkA_mean_fold-change_windows	2.59	5.73	4.094	0.9162	0.9572	1.926	3.657	< 0.00001
204,944 data points			KBM7_clone14_LADs	0.0196	0.919	0.227	0.08167	0.2858			

chr20:18,285,354-45,657,791	0.09728	0.009463	AnkA_mean_fold-change_windows	3.12	7.76	6.074	1.502	1.225	0.2693	5.945	0.208681
55,579 data points			KBM7_clone14_LADs	0.0221	1.55	0.4774	0.196	0.4427			
chr21:8,611,169-10,785,070	0.7295	0.5322	AnkA_mean_fold-change_windows	3.72	5.97	5.406	0.7288	0.8537	0.4025	4.498	< 0.00001
12,413 data points			KBM7_clone14_LADs	0	3.92	2.256	2.394	1.547			
chr22:15,622,801-29,122,188	0.5819	0.3386	AnkA_mean_fold-change_windows	2.82	4.19	3.62	0.1187	0.3445	0.7888	3.384	< 0.00001
76,725 data points			KBM7_clone14_LADs	0	0.823	0.2988	0.06459	0.2541			
chrX:2,860,219-155,768,174	0.4121	0.1699	AnkA_mean_fold-change_windows	2.94	10.6	5.71	2.086	1.444	0.6174	5.038	< 0.00001
1,937,895 data points			KBM7_clone14_LADs	0	4.48	1.088	0.9299	0.9643			
**OVERALL**	**0.2803**	**0.07859**	**AnkA_mean_fold-change_windows**	**2.3**	**18.9**	**5.727**	**3.835**	**1.958**	**0.5821**	**5.226**	**< 0.00001**
**12,042,115 data points**			**KBM7_clone14_LADs**	**0**	**4.7**	**0.8595**	**0.8893**	**0.943**			

[1] Bonferroni correction for α=0.05: 0.002173913
