# Supplementary material for: Genome-Wide Anaplasma phagocytophilum AnkA-DNA Interactions Are Enriched in Intergenic Regions and Gene Promoters and Correlate with Infection-Induced Differential Gene Expression
Source: Front Cell Infect Microbiol. 2016 Sep 20;6:97. doi: 10.3389/fcimb.2016.00097 (PMC5028410; doi:10.3389/fcimb.2016.00097)

Supplemental data

Figure S1. Histogram of AnkA enrichment by chromosome for 3 *ex vivo* neutrophil donor genomic DNA interaction experiments.

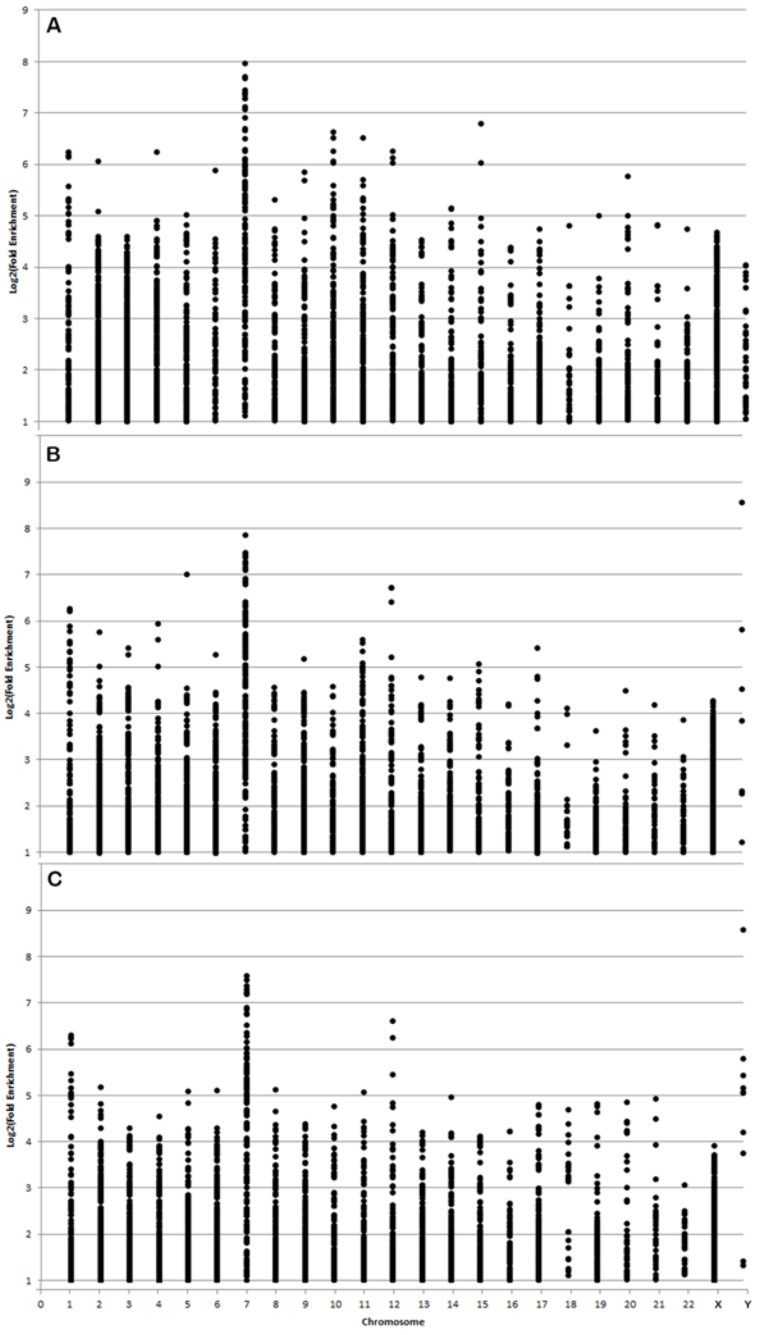

Supplement: Supplementary file 3 [file Image1.PDF]
