# Supplementary material for: Genome-Wide Anaplasma phagocytophilum AnkA-DNA Interactions Are Enriched in Intergenic Regions and Gene Promoters and Correlate with Infection-Induced Differential Gene Expression
Source: Front Cell Infect Microbiol. 2016 Sep 20;6:97. doi: 10.3389/fcimb.2016.00097 (PMC5028410; doi:10.3389/fcimb.2016.00097)

Figure S2. Correlations of AnkA enriched binding sites (A) or AnkA enriched binding sites averaged over ~53 Mbp windows (B) with KMB-7 clone 14 average O/E lamina-associated domains over 22 haploid chromosomes and overall.

## Supplemental Figure 2A

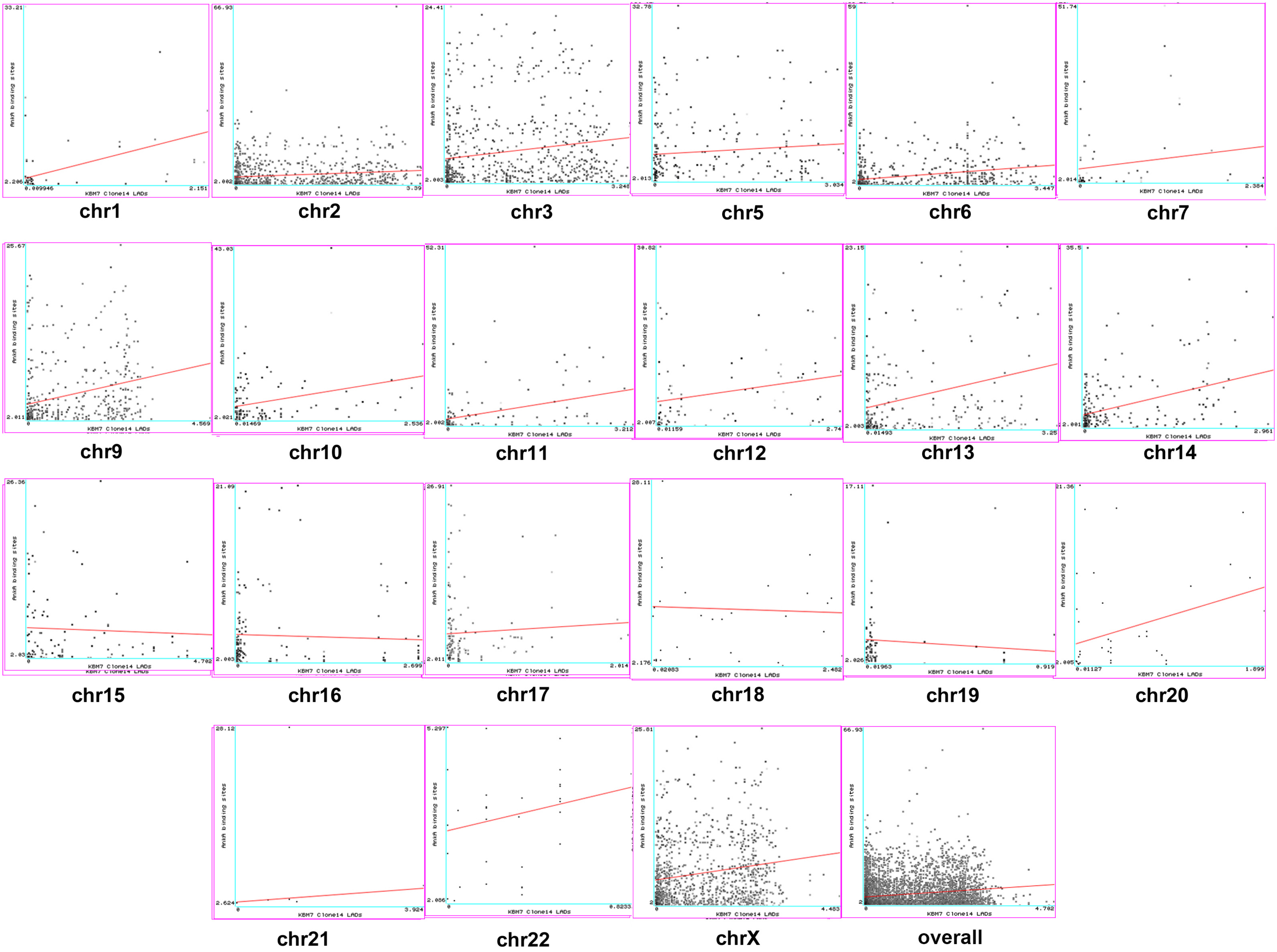

# Supplemental Figure 2B

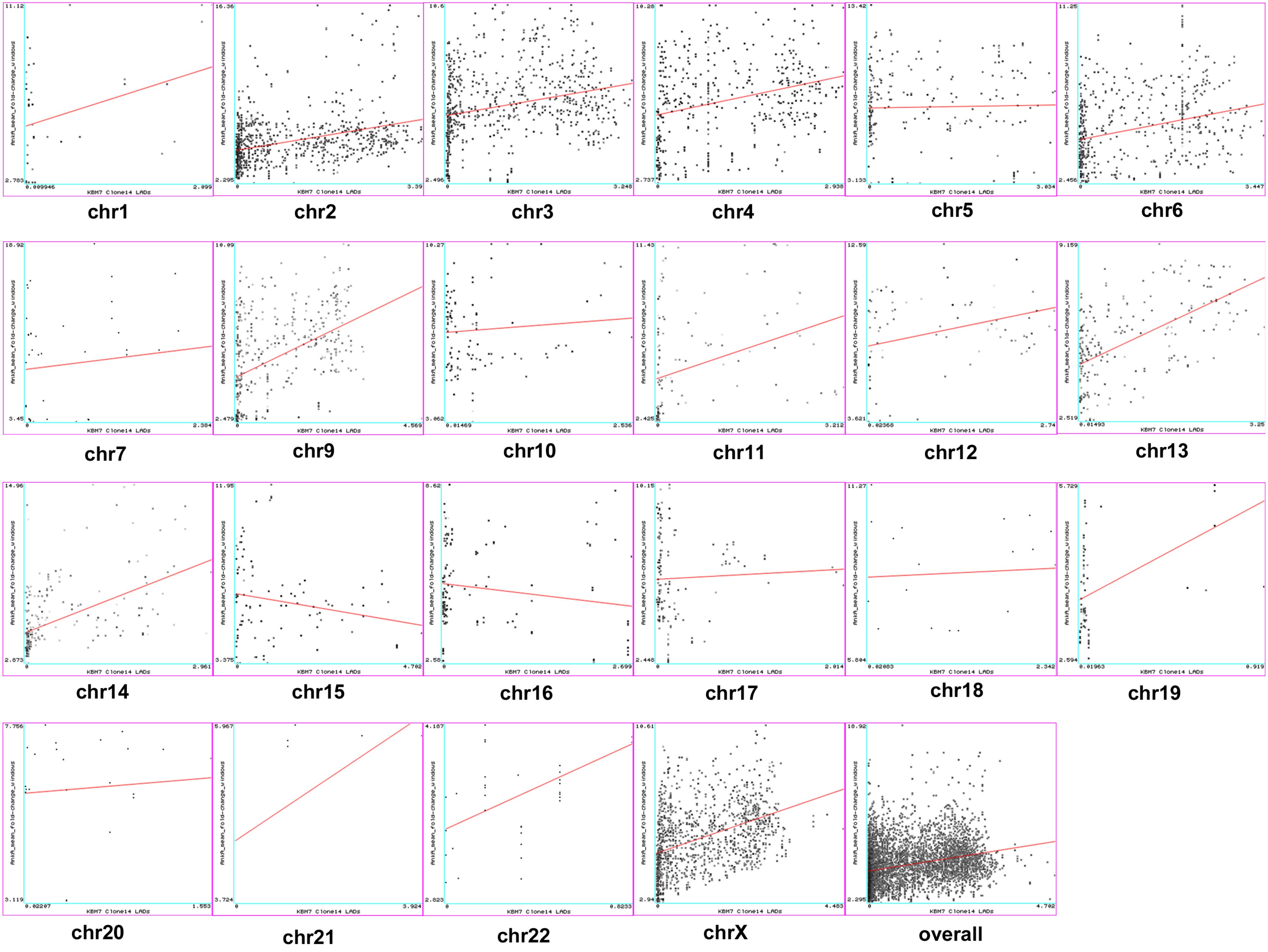

Supplement: Supplementary file 4 [file Image2.pdf]
